# Efficient CRISPR-Cas12f1-Mediated Multiplex Bacterial Genome Editing via Low-Temperature Recovery

**DOI:** 10.4014/jmb.2403.03033

**Published:** 2024-06-14

**Authors:** Se Ra Lim, Hyun Ju Kim, Sang Jun Lee

**Affiliations:** 1 Department of Systems Biotechnology and Institute of Microbiomics, Chung-Ang University, Anseong 17546, Republic of Korea; 2 Nakdonggang National Institute of Biological Resources, Sangju 37242, Republic of Korea

**Keywords:** Nucleotide editing, multiple targets, recovery temperature, AsCas12f1, truncated guide RNA

## Abstract

CRISPR-Cas system is being used as a powerful genome editing tool with developments focused on enhancing its efficiency and accuracy. Recently, the miniature CRISPR-Cas12f1 system, which is small enough to be easily loaded onto various vectors for cellular delivery, has gained attention. In this study, we explored the influence of temperature conditions on multiplex genome editing using CRISPR-Cas12f1 in an *Escherichia coli* model. It was revealed that when two distinct targets in the genome are edited simultaneously, the editing efficiency can be enhanced by allowing cells to recover at a reduced temperature during the editing process. Additionally, employing 3'-end truncated sgRNAs facilitated the simultaneous single-nucleotide level editing of three targets. Our results underscore the potential of optimizing recovery temperature and sgRNA design protocols in developing more effective and precise strategies for multiplex genome editing across various organisms.

## Introduction

CRISPR-Cas system is an immune mechanism of prokaryotes that specifically recognizes and degrades exogenous nucleic acids, genetically protecting the organism [1]. The function of the CRISPR-Cas system is divided between the guide RNA (gRNA) for target nucleic acid recognition and the Cas nuclease for cleavage [1]. This modular system allows for the cleavage of desired nucleotide sequences by modifying the target recognition sequence (TRS) on the gRNA [2], thereby enabling genome editing across various organisms, including microbes [3, 4].

Class 2 CRISPR-Cas systems, featuring a single effector protein, are primarily employed for genome editing. Notably, extensive research has focused on Cas9 derived from *Streptococcus pyogenes* (SpCas9) [5]. However, heterologous expression of the Cas9 protein can lead to cytotoxicity or abnormal growth [6, 7]. Therefore, endogenous CRISPR-Cas systems [8], Cas9 orthologs [9], and Cas12 or Cas13 [10] are being used as editing tools, offering improved compatibility with target cells. Moreover, the large size of the widely used SpCas9 (4.1 kb; 1,368 a.a.) presents challenges, particularly in packaging into viral vectors with limited space [11].

A miniature CRISPR-Cas12f1 system has garnered attention for its potential to address this challenge. Cas12f1 orthologs consist of single polypeptides of around 500 a.a., which are significantly shorter than the length of Cas9 [12]. The Cas12f1 nuclease is known to form a dimer, with each single RuvC domain cutting both strands of the target DNA [13, 14]. The use of Cas12f1 nuclease for single gene editing in the genome has been reported in various organisms, including *Escherichia coli* [15, 16], *Bacillus anthracis* [17], *Klebsiella pneumoniae* [18], mice [19], and humans [20, 21].

Recently, the Cas12f1-mediated simultaneous deletion of two genes was demonstrated in *Streptomyces coelicolor* [22]. However, the application of Cas12f1 for precise multiplex genome editing has not yet been documented. In this study, we attempted single-nucleotide level multiplex genome editing in *E. coli* using the CRISPR-Cas12f1 system. Two strategies—adjusting the cell recovery temperature and modifying the gRNA—were employed, and their impacts on the efficiency and accuracy of multiplex genome editing were assessed.

## Materials and Methods

### Strains and Culture Conditions

The *E. coli* strains used in this study are listed in Table S1. *E. coli* DH5α served as the cloning host for plasmid construction. *E. coli* HL061, engineered with the *cas12f1* gene from *Acidibacillus sulfuroxidans* (*Ascas12f1*) integrated into the araBAD operon, was employed for genome editing experiments. To enhance the intracellular stability of single-stranded mutagenic oligonucleotides, the pHK463 plasmid expressing λ Beta protein was used, as described previously [23]. Each strain was cultured in LB broth (LPS solution, Cat. No. LB-05, Republic of Korea) at either 30 or 37°C, depending on the *ori* of the plasmid. Ampicillin (50 μg/ml) or spectinomycin (75 μg/ml) was added to the media when required. For preparing electrocompetent cells, HL061 was cultured until OD_600nm_ = 0.4. To induce the expression of the λ Beta protein and Cas12f1, L-arabinose was added at a final concentration of 1 mM. Following a 3 h incubation, the cells were harvested, washed twice with 10% glycerol, resuspended, and stored in aliquots at −80°C.

### Construction of Multiple sgRNA Plasmids

The plasmids used in this study are listed in Table S1. The primers used for plasmid construction are listed in Table S2. PCR amplification was performed using KOD FX (Toyobo, Cat. No. TOKFX-101, Japan), while ligation was conducted using Gibson Assembly Master Mix (NEB, Cat. No. E2611, USA). A dual sgRNA plasmid, pSR052, was constructed by inserting an sgRNA cassette downstream of the spectinomycin resistance gene in the single sgRNA plasmid pHL294. The chloramphenicol resistance gene, amplified from pACYC184, was employed to construct pSR076, replacing the streptomycin resistance gene of pSR052. For the generation of pSR053 and triple sgRNA plasmids (pSR078 and pSR082), sets of primers with different TRS overhangs were used to ensure that each PCR product contained either the origin of replication (*ori*) or the antibiotic resistance gene. The sequences of the sgRNA cassettes were verified through Sanger sequencing.

### Multiplex Genome Editing via CRISPR-Cas12f1-Mediated Negative Selection

The sequences of the mutagenic oligonucleotides used in the genome editing experiments are listed in Table S3. Mutagenic oligonucleotides (70mer, 500 pmol) and sgRNA plasmid (200 ng) were simultaneously electroporated into competent cells overexpressing Cas12f1 and λ Beta protein. Electroporation was performed under the following conditions: 25 μF, 200 Ω, 1.8 kV, and 0.1 cm electroporation cuvette (Bio-Rad). Immediately after electroporation, 950 μl of SOC medium was added. The cells were recovered at temperatures of 17, 27, and 37°C, shaking at 180 rpm for durations of 1, 3, 6, 12, and 18 h. Post-recovery, the cells were plated on MacConkey agar (BD Difco, Cat. No. 281810, USA) and incubated at 30°C for 16–30 h until the colony colors developed completely. To observe fermentation phenotypes, D-galactose (0.5%, CAS No. 59-23-4), D-xylose (0.5%, CAS No. 58-86-6), and D-sorbitol (0.5%, CAS No. 50-70-4) were added to the MacConkey agar as needed.

### Assessment of Multiplex Genome Editing Efficiency and Accuracy

To evaluate the in vivo cleavage activity, the transformation efficiencies of pHL308 (sgRNA-deleted plasmid) and a multiple sgRNA plasmid were calculated. The editing efficiency was calculated as the percentage of white colonies to total colonies on MacConkey agar. After conducting three independent experiments, four white colonies were randomly selected to confirm single-nucleotide editing. The target genes were amplified and Sanger sequencing was performed. Successful editing was confirmed only when all target genes were accurately edited in a single colony. The primers used for PCR amplification and Sanger sequencing are listed in Table S2.

## Results

### Designing Multiplex Genome Editing Using the CRISPR-Cas12f1 System

**Fig. 1:**
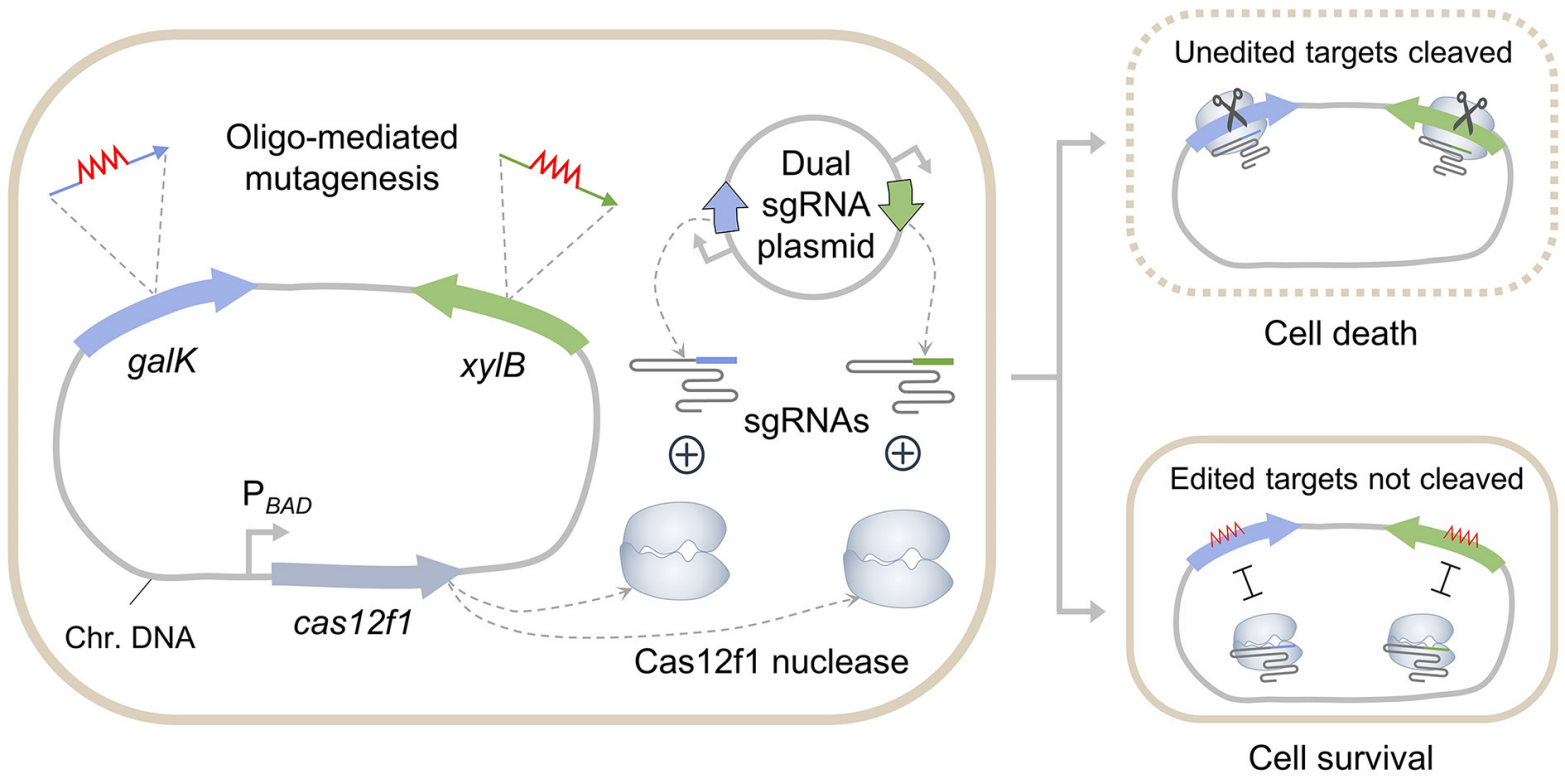
Multiplex genome editing through Cas12f1-mediated negative selection. Mutations are introduced into the *galK* and *xylB* genes by oligonucleotides. sgRNAs are expressed from a dual sgRNA plasmid and form a complex with Cas12f1 nuclease. The scaffold sequences of both sgRNAs are identical and indicated by gray lines. Each sgRNA has a targeting region sequence (TRS) specific to either *galK* or *xylB*. Unedited targets are cleaved by the sgRNA/Cas12f1 complex. However, cells with edited targets are not cleaved, facilitating the identification of edited cells through negative selection.

*galK* and *xylB* genes were targeted for simultaneous editing in the *E. coli* genome. The dual sgRNA plasmid pSR052 was designed to prevent recombination by positioning each sgRNA cassette between the *ori* and the spectinomycin resistance gene. To increase the efficiency of oligonucleotide-directed mutagenesis, λ Beta recombinase was expressed using the pHK463 vector [23]. The expression of the Cas12f1 protein was regulated using the L-arabinose-inducible P*_BAD_* promoter. Cas12f1 was utilized as a negative selection tool to effectively eliminate unedited cells (Fig. 1). Unedited targets were cleaved by the sgRNA/Cas12f1 complex, leading to cell death. Multiplex-edited cells survived and appeared as white colonies on MacConkey agar, as they were unable to use D-galactose and D-xylose as carbon sources.

### Effect of Low-Temperature Recovery on Double Target Editing Efficiency

**Fig. 2:**
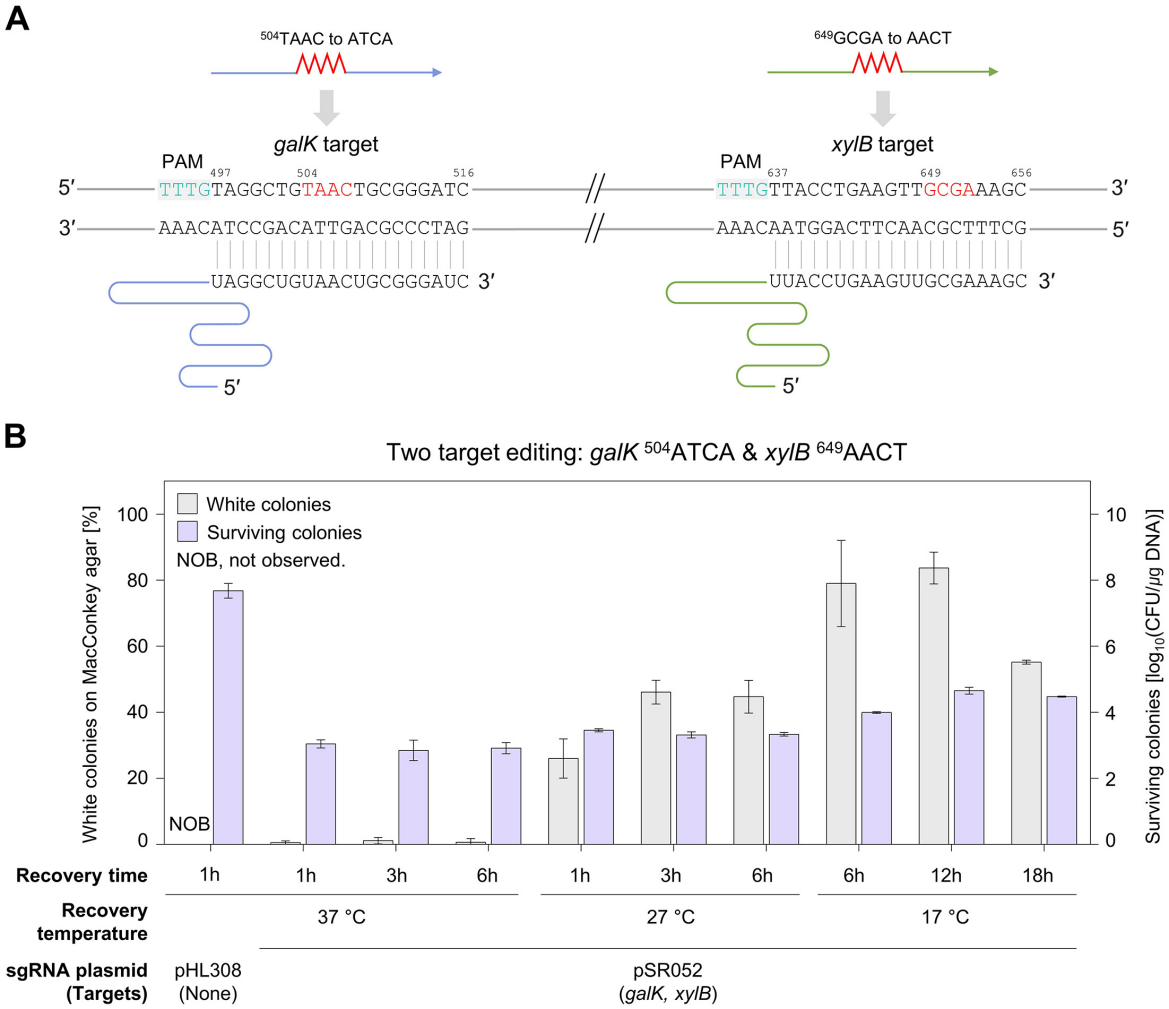
Simultaneous editing of *galK* and *xylB* targets. (**A**) sgRNAs recognizing *galK* and *xylB* target sequences. Mutations are introduced through oligonucleotides, resulting in changes from ^504^TAAC to ATCA for the *galK* target and from ^649^GCGA to AACT for the *xylB* target. Each sgRNA possesses a 20 nt long TRS specific to the *galK* or *xylB* target. Gray boxes indicate PAM sequences and target nucleotides are marked in red. (**B**) Optimization of recovery temperature for multiplex genome editing. Recovery temperatures of 37, 27, and 17°C were investigated, with varying recovery times at each temperature. Gray and purple bars indicate editing efficiency and the number of surviving cells, respectively.

Mutagenic oligonucleotides induce 4 nt substitutions in the *galK* and *xylB* targets, generating stop codons (Fig. 2A). Initially, multiplex genome editing was attempted using the same conditions as in a previous study using CRISPR-Cas9 [24], including recovery at 37 °C. However, white colonies were observed at less than 1%. Furthermore, increasing the recovery time to up to 6 hours did not increase editing efficiency (Fig. 2B). Previous studies have suggested that adjusting the recovery temperature could enhance single-target editing efficiency [25-31]. Therefore, We evaluated multiplex editing efficiency at different recovery temperatures. Recovery at 42°C for 1 or 2 h resulted in low editing efficiencies (≤ 2%) (Fig. S1). However, recovery at 27°C for 3 h resulted in an editing efficiency of 46%. Moreover, the highest editing efficiency (83%) was achieved with a 12-h recovery period at 17°C. The transformation efficiencies of pHL308 (sgRNA-deleted plasmid) (10^8^ CFU/μg DNA) and pSR052 (10^3^–10^4^ CFU/μg DNA) indicated that CRISPR-Cas12f1-mediated negative selection was effective. The survival rate at a recovery temperature of 37°C was at the level of 10^3^ CFU/μg DNA. However, when recovery was performed at the optimal temperature of 17°C, a higher survival rate of 10^4^ CFU/μg DNA was observed. Recovery conditions at lower temperatures contributed to higher editing efficiency and survival rates.

### Simultaneous Triple Target Editing under Low-Temperature Conditions

**Fig. 3:**
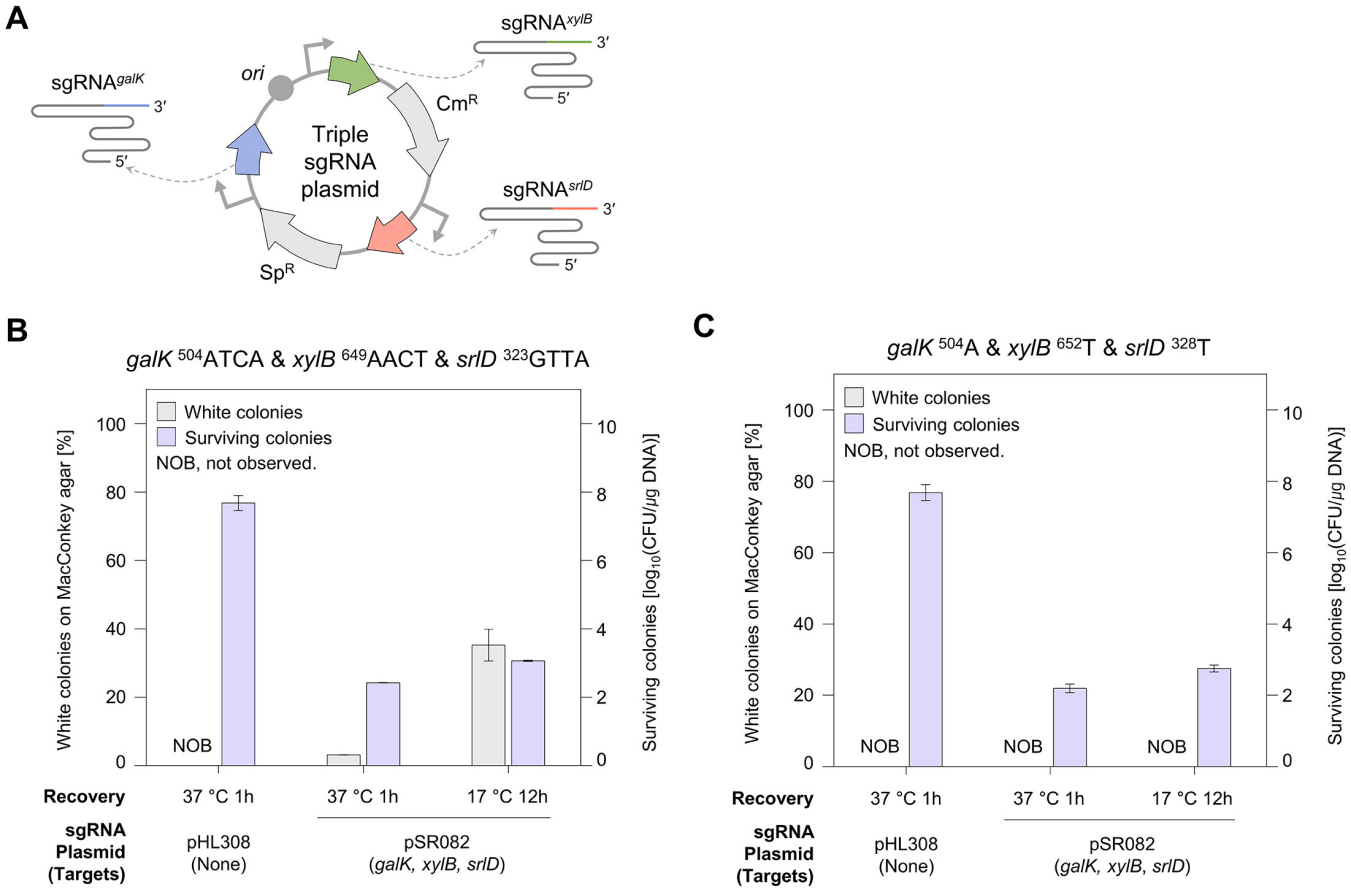
Multiplex three-target genome editing. (**A**) Plasmid expressing three sgRNAs (targeting *galK*, *xylB*, and *srlD*, respectively). The triple sgRNA plasmid was designed to avoid the loss of sgRNAs resulting from recombination between repetitive scaffold sequences. Each sgRNA cassette expresses *galK*-, *xylB*-, and *srlD*-targeting sgRNAs. (**B**) Multiplex 4 nt substitution in the *galK*, *xylB*, and *srlD* genes. The efficiencies of editing *galK* ^504^ATCA, *xylB* ^649^AACT, and *srlD* ^323^GTTA were compared under two recovery conditions: 1 hour at 37°C and 12 hours at 17°C. (**C**) Multiplex single-nucleotide substitution in the *galK*, *xylB*, and *srlD* genes. The efficiency of editing *galK* ^504^A, *xylB* ^652^T, and *srlD* ^328^T was compared under two recovery conditions: 1 h at 37°C and 12 h at 17°C.

We aimed to simultaneously edit three targets (*galK*, *xylB*, and *srlD*) under optimized low-temperature conditions. To achieve this, a triple sgRNA plasmid, pSR082, was designed to stably express three sgRNAs (Fig. 3A). The survival rate of cells transformed with the triple sgRNA plasmid was 10^3^–10^3.5^ CFU/μg DNA, which was slightly lower than that of cells transformed with the dual sgRNA plasmid (pSR052). Recovery at lower temperatures resulted in relatively higher survival rates (Fig. 3B). When editing three targets with a 1-h recovery period at 37°C, the percentage of white colonies obtained was less than 3%. However, the editing efficiency increased to 35% when cells were recovered at 17°C for 12 h. Subsequently, the possibility of achieving single-nucleotide level editing under low-temperature conditions was investigated. Mutagenic oligonucleotides introducing single-nucleotide substitution into each target gene (*galK* ^504^T to A, *xylB* ^652^A to T, and *srlD* ^328^C to T) were transformed. However, white colonies were not observed (Fig. 3C).

### Single-Nucleotide Level Multiplex Genome Editing by 3'-End Truncated sgRNAs

**Fig. 4:**
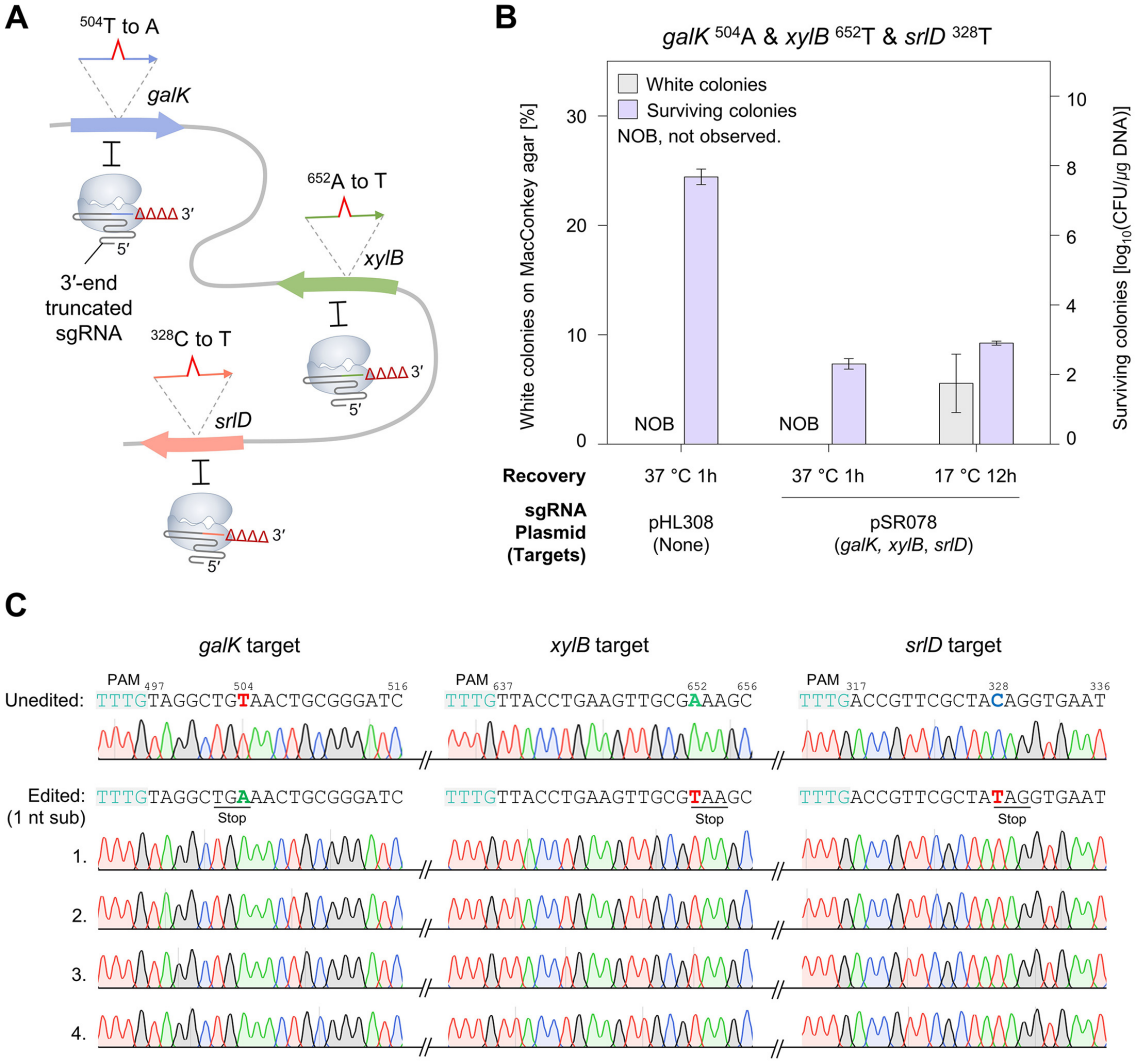
Single-nucleotide level multiplex genome editing using 3'-end truncated sgRNAs. (**A**) A schematic diagram of simultaneous three-target editing. The 3'-end truncated sgRNA/Cas12f1 complexes cleave unedited target sequences while leaving single-nucleotide-substituted sequences intact. Therefore, cells survive only when editing events occur simultaneously at all three targets. (**B**) Multiplex single-nucleotide substitution in the *galK*, *xylB*, and *srlD* genes using 3'-end truncated sgRNAs. The efficiencies of editing *galK* ^504^A, *xylB* ^652^T, and *srlD* ^328^T using truncated sgRNAs were compared under two recovery conditions: 1 h at 37°C and 12 h at 17°C. (**C**) Sanger sequencing analysis of the single-nucleotide-edited cells. Four white colonies were randomly selected and the target sequences of the *galK*, *xylB*, and *srlD* genes were analyzed. The target nucleotides are highlighted in color and bold font.

Achieving single-nucleotide editing is challenging because of the mismatch tolerance of the CRISPR-Cas system [23]. To address this problem, the 3'-end of the sgRNA was maximally truncated, as described previously [15]. The triple sgRNA plasmid pSR078 expresses three 3'-end truncated sgRNAs targeting the *galK*, *xylB*, and *srlD* genes. 3'-end truncated sgRNA/Cas12f1 complexes were incapable of cleaving single-nucleotide-substituted target sequences (Fig. 4A). Thus, the 3'-end truncated sgRNAs could recognize only unedited target sequences, inducing cell death. The CFU was reduced to 10^2^–10^3^ CFU/μg DNA, demonstrating efficient negative selection by the 3'-end truncated sgRNA/Cas12f1 complexes. While single-nucleotide-substituted cells could not be obtained with recovery at 37°C for 1 hour, 5% of white colonies were achieved under low-temperature conditions. Sanger sequencing of the target gene regions in four randomly selected edited white colonies confirmed the precise editing of all target genes without unwanted mutations (Fig. 4C).

## Discussion

The efficiency of individual editing of the *galK* and *xylB* genes using the CRISPR-Cas12f1 system was approximately 80% in our previous study [15]. However, obtaining cells with concurrent modifications in both the *galK* and *xylB* genes was challenging under the same conditions (Fig. 2B). Meanwhile, it has been reported that exposing cells to high temperatures can enhance the efficiency of CRISPR-Cas9-mediated single-target editing in plants [25]. In *Xenopus laevis*, operating Cas9 at a very low temperature resulted in enhanced single-target editing efficiency [31].

Therefore, this study optimized the recovery temperature to enhance editing efficiency and investigated the effects of both high- and low-temperature conditions on genome editing in *E. coli*. Our results demonstrated that employing a low-temperature recovery significantly enhanced the efficiency of multiplex editing of the *galK* and *xylB* genes (Fig. 2B). Furthermore, a high efficiency of 35% was obtained for simultaneous editing at three targets under optimized low-temperature conditions (Fig. 3B).

These findings underscore the significance of recovery temperature in CRISPR-Cas-mediated multiplex genome editing. The enhanced efficiency observed with low-temperature recovery can be attributed to several factors. Recovery, a pivotal process in genome editing, enables cells to heal from genetic alterations, including DNA damage and breaks induced by mutagenesis and Cas nucleases. If a double-strand break occurs at an unedited target locus while another target is being edited, it could lead to cell death. Lowering the temperature slows down DNA replication [32], potentially allowing more time for edits to occur across multiple genomic sites. Additionally, lowering the temperature reduces the rates of enzyme biochemical reactions [33]. Therefore, the reduced target cleavage rate by Cas12f1 at lower temperatures may increase the likelihood of obtaining multiplex-edited cells.

**Fig. 5:**
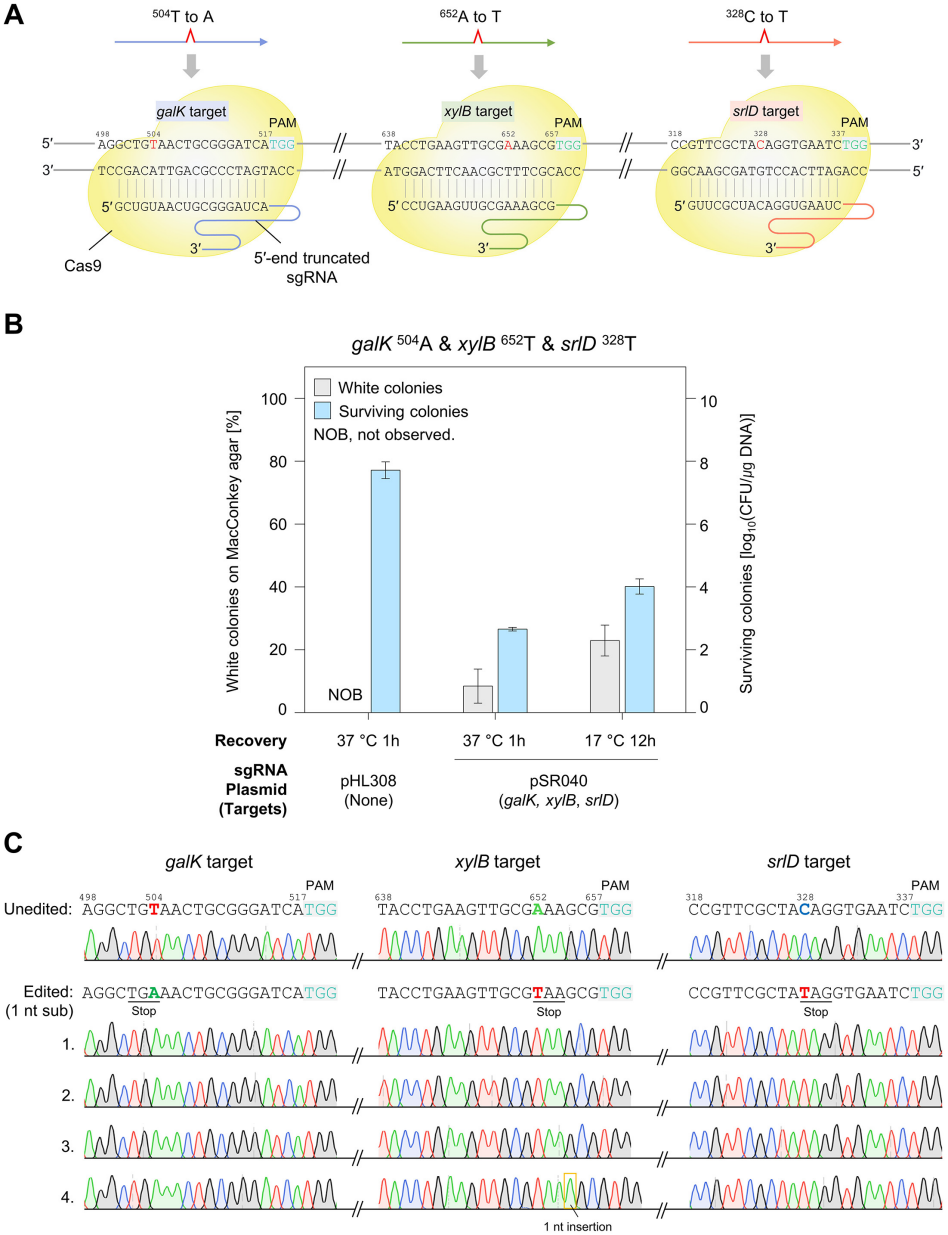
Multiplex genome editing using CRISPR-Cas9 and low-temperature recovery. (**A**) The 5'-end truncated sgRNAs targeting the *galK*, *xylB*, and *srlD* genes. These 5'-end truncated sgRNAs form a complex with Cas9, facilitating the recognition and cleavage of the targets. If a single-nucleotide substitution occurs (*galK* ^504^A, *xylB* ^652^T, and *srlD* ^328^T), the target is not cleaved. (**B**) CRISPR-Cas9-mediated multiplex single-nucleotide substitution in the *galK*, *xylB*, and *srlD* genes using 5'-end truncated sgRNAs and low-temperature recovery. The efficiencies of editing *galK* ^504^A, *xylB* ^652^T, and *srlD* ^328^T using the 5'-end truncated sgRNAs were compared under two recovery conditions: 1 h at 37°C and 12 h at 17°C. (**C**) Sanger sequencing analysis of the single-nucleotide-edited cells. Four white colonies were randomly selected and the target sequences of the *galK*, *xylB*, and *srlD* genes were analyzed. The target nucleotides are highlighted in color and bold font. An undesired mutation is indicated by a yellow box.

The effectiveness of low-temperature recovery in enhancing the efficiency of Cas9-mediated multiplex genome editing was also investigated. Consistent with the targets used in the previous experiment using Cas12f1, the *galK*, *xylB*, and *srlD* genes were targeted for multiplex single-nucleotide substitution (Fig. 5A). Recovery at 37 °C for 1 h resulted in 8% of the colonies being white, while recovery at 17°C for 12 h enhanced the editing efficiency to 23%(Fig. 5B). Sequencing of the *galK*, *xylB*, and *srlD* genes confirmed accurate editing in three out of four white colonies (Fig. 5C). These findings suggest that the low-temperature recovery method might be applicable for multiplex genome editing using various CRISPR-Cas systems in microbial cells.

While 4 nt substitutions were successfully introduced using low-temperature recovery and untruncated sgRNAs (Figs. 2B and 3B), single-nucleotide-edited cells were not obtained (Fig. 3C). Previous studies have reported that using target-mismatched gRNAs [23, 34] or maximally truncated gRNAs [15, 35, 36] could effectively overcome mismatch tolerance and achieve precise genome editing. Therefore, we employed 3'-end truncated sgRNAs to accomplish precise single-nucleotide multiplex genome editing at three different targets (Fig. 4). Moreover, the use of truncated sgRNA enhances target specificity and reduces off-target effects [37].

Sequential CRISPR-Cas-mediated genome editing requires the repeated transformation and removal of multiple plasmids. This one-at-a-time process is laborious and increases the likelihood of additional genetic mutations arising from multiple rounds of cultivation. In contrast, multiplex genome editing saves time and cost by enabling the simultaneous modification of multiple genes to obtain cells with desired genotypes. Overall, this study demonstrates the efficacy of combining low-temperature recovery with truncated sgRNAs for precise Cas12f1-mediated multiplex genome editing, contributing to the advancement of genome editing technologies in the field of microbiology and biotechnology.

## Supplemental Materials

Supplementary data for this paper are available on-line only at http://jmb.or.kr.
