# Supplementary material for: Efficient CRISPR-Cas12f1-Mediated Multiplex Bacterial Genome Editing via Low-Temperature Recovery
Source: J Microbiol Biotechnol. 2024 Jun 14;34(7):1522–9. doi: 10.4014/jmb.2403.03033 (PMC11294644; doi:10.4014/jmb.2403.03033)
Supplement: Supplementary file 1 [file jmb-34-7-1522-supple.pdf]

J. Microbiol. Biotechnol. 2024. 34(7): 1522–1529
https://doi.org/10.4014/jmb.2403.03033

Supplementary Figure and Tables

Efficient CRISPR-Cas12f1-Mediated Multiplex Bacterial Genome Editing via Low-Temperature Recovery

Se Ra Lim[1], Hyun Ju Kim[1,2], and Sang Jun Lee[1]*

[1]Department of Systems Biotechnology and Institute of Microbiomics, Chung-Ang University, Anseong 17546, Republic of Korea

[2]Present address: Nakdonggang National Institute of Biological Resources, Sangju 37242, Republic of Korea

*Corresponding author: sangjlee@cau.ac.kr

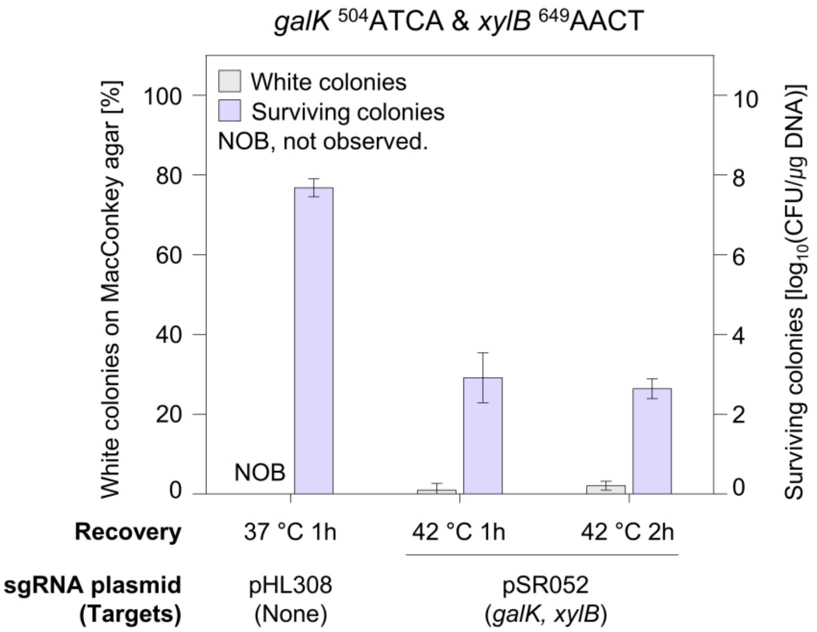

Fig. S1. Multiplex editing efficiency of *galK* and *xylB* at 42 °C. The efficiencies of simultaneously editing the *galK* and *xylB* genes were compared under conditions of 1- or 2-hour recovery at 42 °C.

Table S1. Strains and plasmids used in this study.

Name	Characteristics	Source/reference
**Strain**		
DH5α	*fhuA2 lac(del)U169 phoA glnV44 Φ80′ lacZ(del)M15 gyrA96 recA1 relA1 endA1 thi-1 hsdR17*	Laboratory stock
MG1655	F^- *ilvG rfb*-50 *rph*-1	Laboratory stock
HK1059	MG1655, *araBAD*::P_{BAD}-*cas9*-FRT-Km^R-FRT	[1]
HL061	MG1655, *araBAD*::P_{BAD}-*cas12f1*-FRT-Km^R-FRT	[2]
**Plasmid**		
pACYC184	p15A *ori*, Cm^R, Tc^R	[3]
pHK463	pSC101 *ori*^{ts}, *araC*, λ *bet* gene, Amp^R	[1]
pHL308	pBR322 *ori*, Sp^R	[2]
pHL267	pBR322 *ori*, sgRNA target (^{497}TAGGCTGTAACTGCGGGATC^{516} in *galK*), Sp^R	[2]
pHL294	pBR322 *ori*, sgRNA target (^{637}TTACCTGAAGTTGCGAAAGC^{656} in *xylB*), Sp^R	[2]
pSR040	pBR322 *ori*, Cas9 sgRNA targets (^{500}GCTGTAACTGCGGGATCA^{517} in *galK,* ^{640}CCTGAAGTTGCGAAAGCG^{657} in *xylB*, ^{318}CCGTTCGCTACAGGTGAATC^{337} in *srlD*), Cm^R, Sp^R	[4]
pSR052	pBR322 *ori*, sgRNA targets (^{497}TAGGCTGTAACTGCGGGATC^{516} in *galK*, ^{637}TTACCTGAAGTTGCGAAAGC^{656} in *xylB*), Sp^R	This study
pSR076	pBR322 *ori*, sgRNA targets (^{497}TAGGCTGTAACTGCGGGATC^{516} in *galK*, ^{637}TTACCTGAAGTTGCGAAAGC^{656} in *xylB*), Cm^R	This study
pSR078	pBR322 *ori*, sgRNA targets (^{497}TAGGCTGTAACTGCGG^{512} in *galK*, ^{637}TTACCTGAAGTTGCGA^{652} in *xylB*, ^{317}ACCGTTCGCTACAGGT^{332} in *srlD*), Cm^R, Sp^R	This study
pSR082	pBR322 *ori*, sgRNA targets (^{497}TAGGCTGTAACTGCGGGATC^{516} in *galK*, ^{637}TTACCTGAAGTTGCGAAAGC^{656} in *xylB*, ^{317}ACCGTTCGCTACAGGTGAAT^{336} in *srlD*), Cm^R, Sp^R	This study

1. Lee HJ, Kim HJ, Lee SJ. 2020. CRISPR-Cas9-mediated pinpoint microbial genome editing aided by target-mismatched sgRNAs. *Genome Res*. **30**: 768–775.

2. Lee HJ, Kim HJ, Lee SJ. 2023. Miniature CRISPR-Cas12f1-mediated single-nucleotide microbial genome editing using 3′-truncated sgRNA. *CRISPR J*. **6**: 52–61.

3. Chang AC, Cohen SN. 1978. Construction and characterization of amplifiable multicopy DNA cloning vehicles derived from the P15A cryptic miniplasmid. *J. Bacteriol.* **134**: 1141–1156.

4. Lim SR, Lee HJ, Kim HJ, Lee SJ. 2023. Multiplex single-nucleotide microbial genome editing achieved by CRISPR-Cas9 using 5′-end-truncated sgRNAs. *ACS Synth. Biol.* **12**: 2203–2207.

Table S2. Primers used in this study.

Name	Sequence (5′→3′)	Description
galK_F	CATCAGCGTGACTACCATCCCTGCGTTG	PCR and sequencing primers for *galK* target
galK_R	CCAGCGAGACCTGACCGCAGAACAGGC	
xylB_F	GCTCAACGAGCAGGGTGAGGTGGTTGCTG	PCR and sequencing primers for *xylB* target
xylB_R	GATATCCGCCAGCATCTGACGCCAGTAC	
srlD_F	CTGGGTCATGTCACCCTGCGCTTCGA	PCR and sequencing primers for *srlD* target
srlD_R	GTAGTGGCGGAAGTCCTGTATGCCTG	
pBR322ori_F	GGGAAACGCCTGGTATCTTTATAGTC	Sequencing primers for sgRNA cassette confirmation
Sm_TAA_out	GCAATGGAGCGCCTGCCGGCCCAGTATCAG	
CmR_half_F	GAAACGTTTTCATCGCTCTGGAGTGAATAC	
gRNA_1F	TCGATTGGCTGAGCATATGCTGGATCCTTGACAGCTAGCTC	pSR052 plasmid construction
gRNA_2R	GATCCAGCATATGCTCAGCCAATCGACTGGCGAGCGGCATC	
gRNA_3F	ATCCCTACTCGAGCTCATAAGTTCCTATTCCGAAGTTCCGC	
gRNA_4R	AGGAACTTATGAGCTCGAGTAGGGATAACAGGGTAATAGAT	
CmR_pACYC184_F	TGATCGGCACGTAAGAGGTTCCAACTTTC	pSR076 plasmid construction
CmR_pACYC184_R	TTACGCCCCGCCCTGCCACTCATCGCAG	
pTarget_CmR_R	GGAACCTCTTACGTGCCGATCAACTCGAGTAGGGATAACAGGGTAATAGATCTAAGC	
pTarget_CmR_F	GAGTGGCAGGGCGGGGCGTAAGATGCCGCTCGCCAGTCGATTGGCTGAGC	
SpR_R	CGATACTTCGGCGATCACCGCTTCCCTC	pSR078, pSR082 plasmid construction
pBR322ori_TAA_out	TTCTGTGGATAACCGTATTACCGCCTTTGAGTGAGCTGATA	
pBR322_R	CACGCTGTAGGTATCTCAGTTCGGTG	

Table S2. Primers used in this study (Continued).

Name	Sequence (5′→3′)	Description
galK497_As12f1_16_F	GAACTAGGCTGTAACTGCGGTTTTTTTGAATTCTCTAGAGTCG	pSR078 plasmid construction
galK497_As12f1_16_R	CCGCAGTTACAGCCTAGTTCACACTCCACAAGCTAG	
xylB637_As12f1_16_F	GAACTTACCTGAAGTTGCGATTTTTTTGAATTCTCTAGAGTCG	
xylB637_As12f1_16_R	TCGCAACTTCAGGTAAGTTCACACTCCACAAGCTAG	
srlD317_As12f1_16_F	GAACACCGTTCGCTACAGGTTTTTTTTGAATTCTCTAGAGTC	
srlD317_As12f1_16_R	AAACCTGTAGCGAACGGTGTTCACACTCCACAAGCTAGCTC	
galK497_As12f1_20_F	TAGGCTGTAACTGCGGGATCTTTTTTTGAATTCTCTAGAGTC	pSR082 plasmid construction
galK497_As12f1_20_R	GATCCCGCAGTTACAGCCTAGTTCACACTCCACAAGCTAGC	
xylB637_As12f1_20_F	TTACCTGAAGTTGCGAAAGCTTTTTTTGAATTCTCTAGAGTCG	
xylB637_As12f1_20_R	GCTTTCGCAACTTCAGGTAAGTTCACACTCCACAAGCTAG	
srlD317_As12f1_20_F	ACCGTTCGCTACAGGTGAATTTTTTTTGAATTCTCTAGAGTC	
srlD317_As12f1_20_R	ATTCACCTGTAGCGAACGGTGTTCACACTCCACAAGCTAGC	

Table S3. Mutagenic oligonucleotides used in this study.

Name	Sequence (5′→3′)	Description
galKT504A_70	ACGGTCAGGAAGCAGAAAACCAGTTTGTAGGCTGAAACTGCGGGATCATGGATCAGCTAATTTCCGCGCT	Mutagenic oligonucleotides for *galK* ^{504}T to A substitution
galKTAAC504ATCA_70	CGGTCAGGAAGCAGAAAACCAGTTTGTAGGCTGATCATGCGGGATCATGGATCAGCTAATTTCCGCGCTC	Mutagenic oligonucleotides for *galK* ^{504}TAAC to ATCA substitution
xylBA652T_70	CGAAATTACTGGTGCTTTGTTACCTGAAGTTGCGTAAGCGTGGGGTATGGCGACGGTGCCAGTTGTCGCA	Mutagenic oligonucleotides for *xylB* ^{652}A to T substitution
xylBGCGA649AACT_70	AGCGAAATTACTGGTGCTTTGTTACCTGAAGTTAACTAAGCGTGGGGTATGGCGACGGTGCCAGTTGTCG	Mutagenic oligonucleotides for *xylB* ^{649}GCGA to AACT substitution
srlDC328T_70	CGACTTCCAGCTCGGCGATTTTGACCGTTCGCTATAGGTGAATCTGGTGGGTTATTTCCTGTGTGCGCGT	Mutagenic oligonucleotides for *srlD* ^{328}C to T substitution
srlDCGCT323GTTA_70	TCAGCGACTTCCAGCTCGGCGATTTTGACCGTTGTTAACAGGTGAATCTGGTGGGTTATTTCCTGTGTGC	Mutagenic oligonucleotides for *srlD* ^{323}CGCT to GTTA substitution

- Underlined nucleotides are the targets for nucleotide substitution.
